# Multi-Omics Analysis Unravels the Impact of Stool Sample Logistics on Metabolites and Microbial Composition

**DOI:** 10.3390/microorganisms12101998

**Published:** 2024-09-30

**Authors:** Jannike L. Krause, Beatrice Engelmann, David J. D. Lallinger, Ulrike Rolle-Kampczyk, Martin von Bergen, Hyun-Dong Chang

**Affiliations:** 1 German Rheumatism Research Center Berlin, A Leibniz Institute—DRFZ, Schwiete Laboratory for Microbiota and Inflammation, 10117 Berlin, Germany; david.lallinger@gmail.com (D.J.D.L.); chang@drfz.de (H.-D.C.); 2 Helmholtz-Centre for Environmental Research—UFZ, Department of Molecular Toxicology, 04318 Leipzig, Germany; beatrice.engelmann@ufz.de (B.E.); ulrike.rolle-kampczyk@ufz.de (U.R.-K.); martin.vonbergen@ufz.de (M.v.B.); 3 Institute of Biochemistry, Faculty of Biosciences, Pharmacy and Psychology, University of Leipzig, 04103 Leipzig, Germany; 4 German Centre for Integrative Biodiversity Research (iDiv) Halle-Jena-Leipzig, 04103 Leipzig, Germany; 5 Department for Cytometry, Institute of Biotechnology, Technical University Berlin, 10115 Berlin, Germany

**Keywords:** faecal samples, intestinal microbiota, storage conditions, untargeted metabolomics, short-chain fatty acids, microbiota flow cytometry, 16S rRNA sequencing

## Abstract

Human health and the human microbiome are inevitably intertwined, increasing their relevance in clinical research. However, the collection, transportation and storage of faecal samples may introduce bias due to methodological differences, especially since postal shipping is a common practise in large-scale clinical cohort studies. Using four different Omics layer, we determined the structural (16S rRNA sequencing, cytometric microbiota profiling) and functional integrity (SCFAs, global metabolome) of the microbiota in relation to different easy-to-handle conditions. These conditions were storage at −20 °C, −20 °C as glycerol stock, 4 °C and room temperature with and without oxygen exposure for a maximum of one week. Storage time affected the microbiota on all Omics levels. However, the magnitude was donor-dependent, highlighting the need for purpose-optimized sample collection in clinical multi-donor studies. The effects of oxygen exposure were negligible for all analyses. At ambient temperature, SCFA and compositional profiles were stable for 24 h and 48 h, respectively, while at 4 °C, SCFA profiles were maintained for 48 h. The global metabolome was highly susceptible, already changing at 24 h in non-frozen conditions. Thus, faecal microbiota was best preserved on all levels when transported as a native sample frozen within 24 h, leading to the least biased outcomes in the analysis. We conclude that the immediate freezing of native stool samples for transportation to the lab is best suited for planned multi-Omics analyses that include metabolomics to extend standard sequencing approaches.

## 1. Introduction

Research focussing on the human microbiota, particularly the intestinal microbiota, has gained broad interest over the past 20 years. One reason for this is the acknowledgement of the microbiota as an integral, essential part of the human body. Stool has thus emerged as a valuable specimen alternative to blood or urine.

Despite compositional differences among individuals, the microbiota have to provide core functions, e.g., biosynthesis of vitamins, short-chain fatty acids, secondary bile acids or enzymes involved in digestion, as well as favouring gut homeostasis by maintaining epithelial integrity, educating and modulating the immune system and protecting against pathogenic species [1,2]. Mapped at the pathway level, these microbial core functions seem homogenous across healthy individuals [3].

The metabolome has widely been recognized as an accurate reflection of the bacterial phenotype, and metabolites have been shown to mediate host–microbiota interactions in the gut and extra-intestinal, distant body sites [4,5]. Thus, a functional characterization of the microbiota may help to better understand how the microbiota differs in health and disease. The metabolome has recently become a frequent focus in research, aiming at advancing disease diagnosis and prognosis, as well as unravelling the mechanism behind pathologies [6,7]. However, one of the key challenges in utilizing stool samples for such research lies in the stability of the faecal metabolome. Variability in sample preservation, ongoing biochemical reactions and external factors can significantly impact metabolite integrity, thereby complicating biomarker discovery and highlighting the need for optimized sample logistics.

Reflecting the broad interest in understanding microbial dysbiosis on a taxonomic level, e.g., using 16S rRNA sequencing or shotgun metagenomics, various studies investigated the impact of stool sample logistics and preservation in the past [8,9,10,11,12]. Changes in microbiota composition, commonly termed dysbiosis, are associated with a variety of different diseases. These shifts generally comprise a reduction in microbial diversity [13,14], a bloom of pathobionts, a loss of beneficial bacteria [15] or changes in microbial functionality [16]. The latter remains insufficiently investigated [8,17], with a limited number of studies focusing on microbial metabolomics [18,19,20]. For metabolomics and sequencing-based methods, the addition of preservatives does not hinder downstream analysis [18,21,22]. But methods relying on sample integrity such as metaproteomics, cytomics or culturomics cannot be applied, suggesting that native stool sample collection is the appropriate procedure in large clinical cohort studies.

Thus, we aimed to define optimal parameters for sample logistics, which allow for the preservation of a sample and only marginally impact the microbiota. The simulated storage conditions utilized in our study were selected for unsupervised home sampling and transportation at low costs, being the most common in clinical trials and research studies and being the preferred method by participants [23]. We simulated eight different conditions in which samples were maintained at different temperatures in the presence and absence of oxygen for up to one week and compared them to the “gold standard” of immediate freezing at −80 °C [18,24]. Using four different Omics layers, we determined structural changes by 16S rRNA sequencing and cytometric microbiota profiling. Functional integrity was assessed via SCFA targeted profiling and untargeted metabolomics for characterizing small molecules originating from both the microbiota and host, as well as diet and external exposures.

## 2. Methods

### 2.1. Simulation of Stool Sample Logistics Under Different Conditions

**Figure 1:**
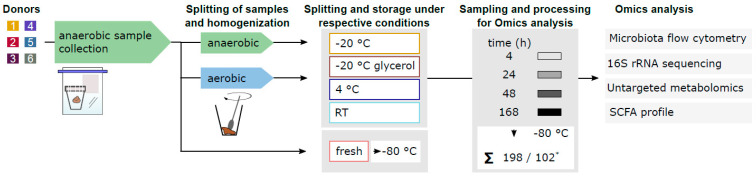
The experimental procedure for simulating sample logistics under different conditions. Samples were collected under anaerobic conditions. The samples were split into two and homogenized in an anaerobic or aerobic atmosphere. From the anaerobic sample, an aliquot was taken for immediate storage at −80 °C as reference. The homogenized samples were then stored at −20 °C, 4 °C and room temperature (RT) or at −20 °C with glycerol as cryoprotectant both under anaerobic and aerobic conditions for 4 h, 24 h, 48 h and 168 h. After all samples were collected, the samples were analysed using microbiota flow cytometry and 16S rRNA sequencing targeting different levels of microbiota composition and using untargeted metabolomics and SCFA profiling to unravel functional changes. * 102 samples for 16S rRNA sequencing, 198 for other Omics.

The stool of six healthy donors (two female and four male donors, aged between 24 and 44) was collected under the approval of the local ethics committee of the Charité Berlin (reference: EA4/014/20; EA2/113/20), and the donors gave written consent. The donors had no antibiotic treatment three months prior to this experiment, no chronic disease and were not on medication. The stool samples were put under anaerobic conditions (Anaerocult P, Oxoid, Hampshire, UK) directly after defaecation and transported to the laboratory within 30 min. There, the stool samples were transferred into an anaerobic chamber (Coy laboratories, Grass Lake, MI, USA) for homogenization. As a fresh reference sample, 100 mg homogenized stool was directly processed anaerobically for the respective analysis, i.e., metabolomics, microbiota profiling and 16S rRNA sequencing, and then snap-frozen in liquid nitrogen for subsequent storage at −80 °C. The remaining homogenized stool was split in two equal parts for (i) anaerobic processing and (ii) aerobic sample processing and was again homogenized. Each sample was split into four parts to simulate the respective storage conditions, i.e., 1. native at −20 °C, 2. 1:10 diluted −20 °C in 12.5% glycerol in PBS, 3. native at 4 °C and 4. native at room temperature (RT). The selected conditions mirror those of the samples frozen in the absence (1.) or presence (2.) of glycerol under faecal microbiota transplantation conditions [25,26], chilled (3.) and unchilled (4.) storage and subsequent transportation to the laboratory. All anaerobic stool samples were kept under anaerobic atmosphere and were stored like their aerobic counterparts. The experimental procedure is visualized in Figure 1.

After 4 h, 24 h, 48 h and 168 h, 100 mg stool or 1 mL 1:10 diluted stool in 12.5% glycerol were aliquoted anaerobically or aerobically, respectively. For metabolomics, the stool samples were snap-frozen in liquid nitrogen and transferred to −80 °C prior to metabolite extraction. Subsequently, 100 mg of each faecal sample was mixed with 500 µL acetonitrile (ACN)/water (1:1, *v*/*v*) and homogenized using a TissueLyser II (30 Hz, 10 min; Retsch Qiagen, Hilden, Germany). After centrifugation (2 min, 14,000 rpm), 100 µL was used for untargeted metabolomics, and 38 µL was used for a further derivatization of short-chain fatty acids. For microbiota flow cytometry and 16S rRNA sequencing, undiluted stool aliquots (conditions 1., 3. and 4.) were diluted 1:10 in PBS or pre-reduced PBS, respectively. All diluted stool samples were filtered by 30 µM mesh (MACS SmartStrainer, Miltenyi Biotech, Bergisch Gladbach, Germany). A total of 10 µL of filtered stool was utilized for DNA extraction, and the remaining diluted stool samples were prepared for microbiota flow cytometry.

Once all samples for all modelled storage conditions were collected, we processed the samples at once and analysed them using microbiota flow cytometry, 16S rRNA sequencing, untargeted metabolomics and SCFA profiling (n = 198, n = 102 for 16S analysis).

### 2.2. Untargeted Metabolomics

For the second extraction step, 100 µL of faecal supernatants was mixed with 500 µL methanol/ACN/water (2:3:1, *v*/*v*/*v*) followed by vortexing and sonication. After centrifugation, 100 µL of the supernatant was dried in a SpeedVac^TM^ vacuum concentrator (Eppendorf, Hamburg, Germany). The extracts were resuspended in 100 µL 1% ACN and 0.1% formic acid in water. For LC-MS/MS analysis, 10 µL of each sample was injected onto a UPLC system coupled on-line with a 6546 UHD Accurate-Mass QTOF (both Agilent Technologies, Santa Clara, CA, USA). Extracts were loaded on a UHPLC guard (ZORBAX RRHD Eclipse Plus C18, 1.8 µm, 2.1 mm, Waters, Milford, MA, USA), and separation was achieved on a C18 column (ZORBAX RRHD Eclipse Plus C18, 1.8 µm, 2.1 × 100 mm, Waters). The flow rate was set constant at 0.3 mL/min using a binary solvent system of A (0.1% formic acid in water) and B (0.1% formic acid in ACN). The gradient was as follows: 0–5 min 1% B, 5.1–20 min 1–100% B and 20.1–25 min 1% B. Samples were acquired in negative ionization mode only and run in randomized order. The QTOF was set up in centroid mode with a scan range of 50–1000 m/z. Fragmentation was obtained using a Top2-method. The exclusion time after two same spectra per cycle was 0.2 min.

The obtained raw data (.d-files) were imported into Progenesis QI^®^ software (version 2.1, Waters Corporation, Milford, MA, USA). All samples were processed together in a generic workflow. The adduct ions involved [M-H], [M-H_2_O-H] and [M-H+ACN] for the ionization mode. Chromatograms were aligned in the t_R_ direction based on a reference chromatogram automatically chosen from the data set. After peak detection, only peaks with MS and MS/MS information were used for the subsequent database search. Using the built-in ChemSpider plug-in, the faecal metabolome database (6738 compounds), *E. coli* metabolome database (755 compounds) and KEGG database (19,090 compounds) were used for identification. Precursor and fragment mass tolerance were set to 10 ppm. The resulting feature matrix was exported, and features without any putative identification were removed, as well as features having higher peak areas in solvent blank samples than the mean peak area in the data set. Progenesis and fragment score were set to ≥40 and 5, respectively.

### 2.3. Short-Chain Fatty Acid Analysis

SCFA derivatization was conducted as previously described in a study by Han et al. [27]. In brief, samples were mixed with ACN to a final concentration of 50%. Subsequently, SCFAs were derivatized using 200 mM 3-nitrophenylhydrazine and 120 mM N-(3-dimethylaminopropyl)-N′-ethylcarbodiimide hydrochloride in pyridine. The derivatized compounds were then diluted in 10% ACN. Samples were injected onto a RSLC UltiMate 3000^®^ system (Thermo Fisher, Waltham, MA, USA) coupled on-line with a QTRAP 5500^®^ mass spectrometer (Sciex, Framingham, MA, USA). The chromatographic separation of SCFAs was achieved using an ACQUITY UPLC BEH C18 column (2.1 × 100 mm, 1.7 µm, Waters, Milford, MA, USA) with a matching pre-column. LC was run at a constant flow rate of 0.35 mL/min with a binary solvent system (A: 0.01% formic acid in water and B: 0.01% formic acid in ACN). A scheduled MRM method with specific transitions for every SCFA was conducted for identification and quantification. Data acquisition and peak integration were performed using the Analyst^®^ software (Version 1.7.1), and samples were measured in randomized order. The absolute quantification of SCFAs was conducted using external calibration curves for each compound measured in the beginning, middle and end of the batch.

### 2.4. DNA Extraction and 16S rRNA Sequencing

As described previously [28], 10 µL 1:10 diluted stool was mixed with 30 volumes of sterile 10% Chelex (*w*/*v*) solution. For cell disruption, samples were incubated at 95 °C for 45 min and 1000 rpm shaking (ThermoMixer, Eppendorf, Hamburg, Germany). To remove cell debris, the suspension was centrifuged for 3 min at 13,000× *g*. The DNA-containing supernatant was transferred into a fresh, sterile tube and stored at −20 °C. Since it was already shown that the taxonomic composition, determined by 16S rRNA sequencing or metagenomics, is relatively robust against sample logistic conditions [10,29], we only analysed the 24 h and the 168 h samples by 16S rRNA sequencing.

#### 2.4.1. 16S Library Preparation

In brief, the V3–V4 region of the bacterial 16S gene was amplified using barcoded bacterial primers V3 forward: CCTACGGGNGGCWGCAG and V4 reverse: GACTACHVGGGTATCTAATCC [30] according to the Illumina protocol for 16S library preparation. V3–V4 amplification was performed using 12.5 µL 2× KAPA HiFi HotStart ReadyMix (Roche Applied Science, Penzberg, Germany), 2.5 µL genomic DNA adjusted to 5 ng/µL and 5 µL V3 forward and V4 reverse primer at 1 µM concentration. After initial denaturation (3 min, 95 °C), 35 cycles of 30 s 95 °C denaturation, 55 °C annealing and 72 °C elongation were performed. Amplicon size was checked in a 1% agarose gel. Amplicons were cleaned using AMPure XP beads (Beckman Coulter, Krefeld, Germany) according to the Illumina protocol. Depending on amplicon band intensity, a minimum of 5 µL amplicon DNA was used as the template in index PCR. Index PCR was conducted using the Nextera XT v2 Index kit C (Illumina, San Diego, CA, USA), together with 5 µL of each index primer and 25 µL 2× KAPA HiFi HotStart ReadyMix in a total volume of 50 µL, with initial denaturation (3 min, 95 °C) during eight cycles of 30 s at 95 °C, 30 s at 55 °C and 30 at 72 °C. The indexed amplicons were purified with AMPure XP beads and quality- and size-checked on a Fragment Analyzer 5200 (Agilent Technologies, Santa Clara, CA, USA). Amplicon concentrations were quantified fluorometrically (Qubit, Invitrogen, Waltham, MA, USA) and subsequently pooled in equimolar concentrations to 4 nM. A total of 5 pM of the denatured pool was loaded and sequenced on an Illumina MiSeq instrument ((Illumina, San Diego, CA, USA).

#### 2.4.2. 16S rRNA Sequence Processing

The obtained paired-end reads were filtered and trimmed using Trimmomactic (version 0.39) [31]. 5′ bases below a quality threshold of 35 were trimmed, and 3′ bases with a quality threshold of 5 were trimmed. Reads shorter than 180 bases were filtered out. The remaining reads were processed using the DADA2 pipeline (version 1.26) in R [32]. Forward and reverse reads were removed when they were shorter than 260 bp or 210 bp, respectively, and reads with a minimum quality score of 12 were filtered out. Error rate estimation was performed with the maximum possible number of bases. Denoised forward and reverse reads were merged with default settings. Taxonomy was assigned using the Silva database v138.1.

After taxonomic annotation, data were processed using the *phyloseq* package [33]. For statistical analyses, samples were rarefied to obtain an equal sample depth of 15,000 reads/sample (Supplementary Figure S1). To compare the alpha diversity per sample, the Shannon index [34] and the number of observed amplicon sequence variants (ASVs) were compared. For further analysis, low abundant ASVs with a prevalence threshold of 5% across samples were excluded using the *phyloseq_filter_prevalence* function from the *metagMisc* package (version 0.5.0). Taxonomic binning was conducted with the help of the Rhea package [35] prior to data analysis and statistics.

### 2.5. Microbiota Flow Cytometry

The optical density (OD) of the filtered stool was determined spectroscopically at 620 nm (Multiskan FC, Thermo Fisher Scientific, Waltham, MA, USA). The stool filtrates were diluted to 1.6 OD/mL in 25% glycerol in LB medium, snap-frozen and transferred to −80 °C. For cytometric analysis, 25 µL of the 1.6 OD/mL glycerol stock (=0.04 OD) as well as a pool of all samples per donor was washed with 1 mL 0.22 µM sterile-filtered PBS and centrifuged at 5000× *g* for 10 min at 4 °C. The bacteria were stained in 100 µL 5 µM Hoechst (Thermo Fisher Scientific, Waltham, MA, USA) for 30 min, and the pooled samples remained unstained by adding 100 µL PBS. Residual Hoechst solution was removed by adding 900 µL sterile-filtered PBS followed by centrifugation (5000× *g*, 10 min, 4 °C). The bacteria pellet was suspended in 1 mL sterile-filtered PBS. Prior to acquisition, 1 µL Bright Blue calibration beads were added as size reference. Samples were diluted to an event rate of ~10,000 events/s, and 300,000 Hoechst positive events were acquired using a BD Influx instrument (BD Biosciences, Franklin Lakes, NJ, USA), resulting in single-cell resolved microbiota profiles. Cytometric profiles were analysed in the FlowJo (version 10.8.1) software. There, instrument noise and debris were excluded, and intact bacterial cells were defined. From these, Hoechst-positive events were selected and were gated with a grid to extract relative gate abundances to compare samples for each donor and across donors (Supplementary Figure S2).

### 2.6. Data Analysis and Statistics

The calculation of statistics and visualization of data were conducted in R version 4.1.1 [36]. Differences in community composition (beta diversity) are based on rarefied and prevalence-filtered genus abundances, absolute and relative SCFA abundances, cytometric relative gate abundances and the relative abundance of putatively identified metabolites (feature matrix). Briefly, Bray–Curtis distances were extracted during non-metric multi-dimensional scaling (NMDS) using the *metaMDS* function from the *vegan* package in R (version 2.6) [37,38]. When BC similarity was utilized, the similarity was calculated as BC similarity = 1 − BC distance. Further, intra-individual BC similarity was represented by the mean BC similarity of all samples per donor to the respective fresh sample. Inter-individual BC similarity was calculated as the mean BC of all samples to the fresh sample of all other donors. Differences between groups were calculated using the permutational multi-variate analysis of variance (PERMANOVA) function from the *vegan* package in R (version 2.6) [37], and individual group comparisons were calculated using the *pairwise*.*adonis2* function (version 0.4). For the significance testing of individual variables, a Kruskal–Wallis group test followed by a post hoc pairwise Dunn test from the *rstatix* package (version 0.7.2) was applied, if not stated differently. Cluster dendrograms were calculated with *heatmap.2* (version 3.1.3.1) and correlations with the *stat_cor* function from the *ggpubr* package (version 0.6.0). Correlations were conducted using the Kendall method to handle non-normal distributed data. If not stated otherwise, figures were generated using *ggplot2* (version 3.5.1) [39].

## 3. Results

To understand the impact of storage conditions (temperature, glycerol preservation during freezing) and oxygen exposure over time, we mimicked eight different sample logistic scenarios. To determine microbial composition, we profiled the structure of the microbial communities with microbiota flow cytometry (MFC) and the taxonomic composition by 16S rRNA sequencing. With targeted SCFA and untargeted global metabolomics, we characterized the effects of sample logistics on the microbial metabolome.

### 3.1. The High Inter-Donor Variability in Faecal Microbiota Composition and Functionality Masks the Potential Effects of Sample Logistics

**Figure 2:**
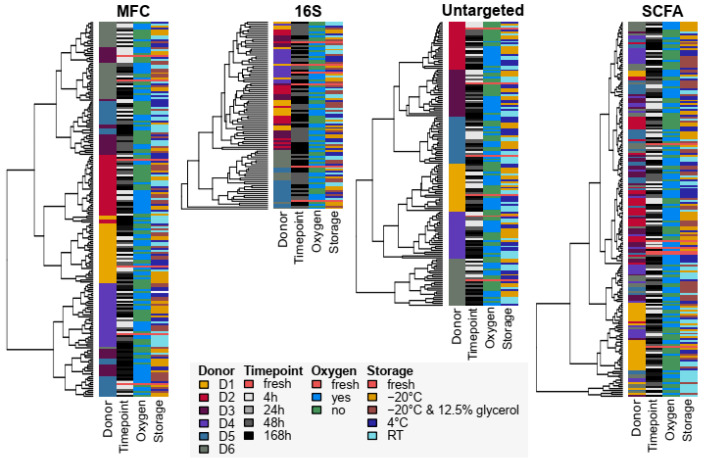
Data visualization of multi-variate Omics data using hierarchical clustering. Hierarchical clustering analyses were performed with all microbiota samples across conditions and time points combined in multi-variate data set. To visualize potential clustering according to donor, time point, oxygen exposure or storage, respective conditions were visualized next to cluster dendrogram.

For a general overview, we performed clustering analyses based on compositional and functional microbiota profiles across all Omics with resolution for donor, time point, oxygen exposure and storage condition (Figure 2).

Glycerol-stored samples were excluded from the clustering analysis for untargeted metabolomics due to severe differences in the base peak chromatograms (Supplementary Figure S3). Hierarchical clustering revealed donor-specific segregation, with no clustering by storage, time or oxygen exposure (Figure 2).

**Figure 3:**
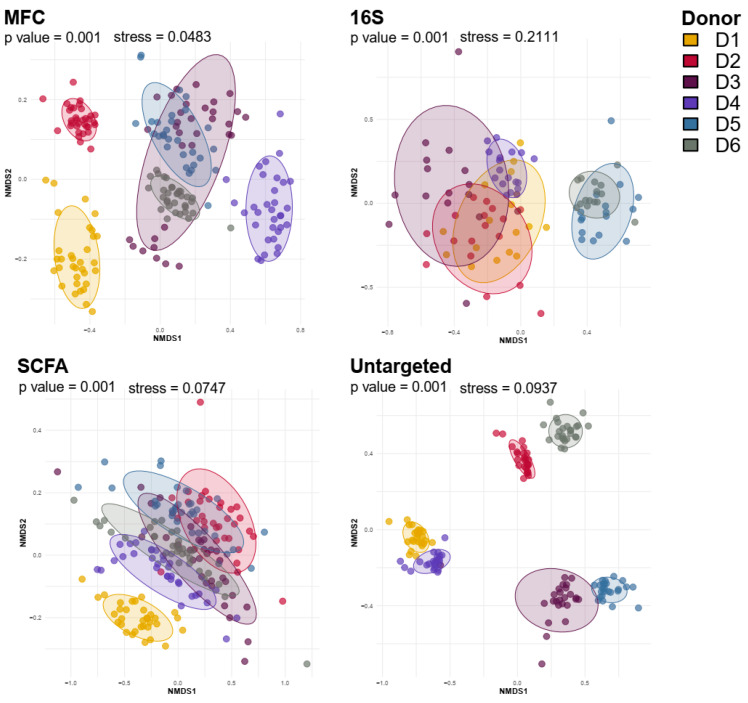
Data visualization of multi-variate Omics data using non-metric dimensional clustering (NMDS). NMDS analyses were performed with all microbiota samples across conditions and time points combined in multi-variate data set using Bray–Curtis distances. Colouring by donor.

We then utilized non-metric dimensional scaling (NMDS) analysis to determine the clustering of samples due to the individuals (Figure 3).

Donor-specific clustering was evident in the cytometric microbiota analysis (MFC) and untargeted metabolomics, indicating a higher donor segregation of microbiota profiles in MFC and global metabolomics compared to that in 16S rRNA profiling and SCFA analysis, which showed more cluster overlap between donors.

**Figure 4:**
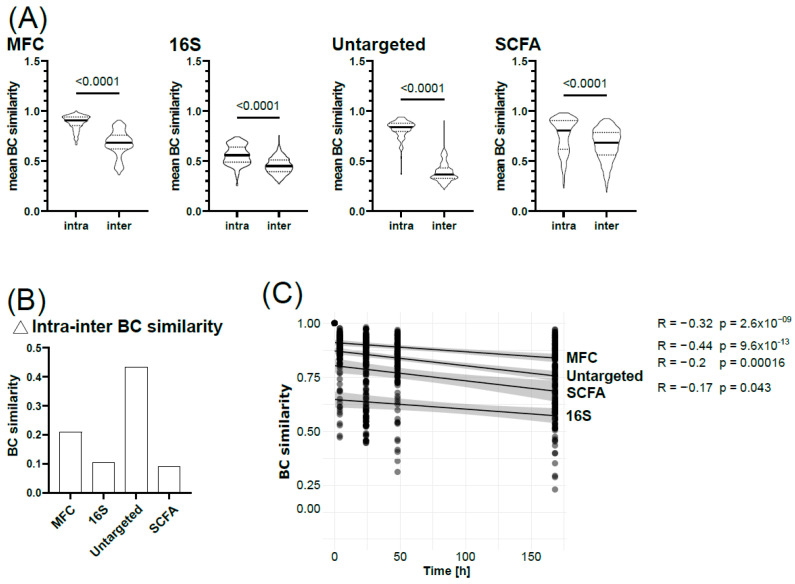
Bray–Curtis similarity analyses. (**A**) Comparison of intra- and inter-individual Bray–Curtis (BC) similarity across all Omics. Group comparison with Student’s *t*-test. (**B**) Difference between intra- and inter-individual BC similarities. (**C**) Correlation of BC similarity and time per Omics data set using Kendall correlation for non-parametric data.

To confirm the donor individuality observed within the different Omics techniques, we extracted Bray–Curtis (BC) distances during NMDS calculation and quantified the intra- and inter-individual similarity of the microbial profiles (Figure 4).

Consistent across all Omics data sets, the intra-individual BC similarity was significantly higher compared to inter-individual BC similarities (Figure 4A), suggesting that sample logistics at all conditions tested within this study preserved the individuality of the microbiota. The difference in inter- and intra-individual BC similarity was higher in analyses with highly individual clustering, i.e., MFC and untargeted metabolomics, compared to those with more overlapping profiles, i.e., SCFA and genus-level profiles (Figure 4B). However, BC similarity decreased over time, regardless of storage conditions, indicating that microbiota composition and function change with time, particularly for untargeted metabolomics (R = −0.44) and MFC (R = −0.32), while SCFAs (R = −0.2) and 16S rRNA (R = −0.17) were less affected (Figure 4C). After 4 h, all profiles had a significantly lower BC similarity to their corresponding fresh microbiota (Supplementary Figure S4). When comparing the respective profiles against the 4 h time point (Supplementary Figure S4, asterisks in brackets), we can confirm that an increased storage time significantly impacts the global metabolome profile and single-cell resolved microbiota profiles, while SCFAs and 16S rRNA profiles remain relatively stable.

Due to the very high donor individuality observed in the initial clustering, we analysed sample logistic-dependent changes on the individual level (Supplementary Figure S5). And indeed, the impact of sample logistics on the donors’ microbiota varied significantly.

### 3.2. SCFA Concentrations Increase over Time Under Non-Frozen Conditions, Especially When Kept Anaerobic

**Figure 5:**
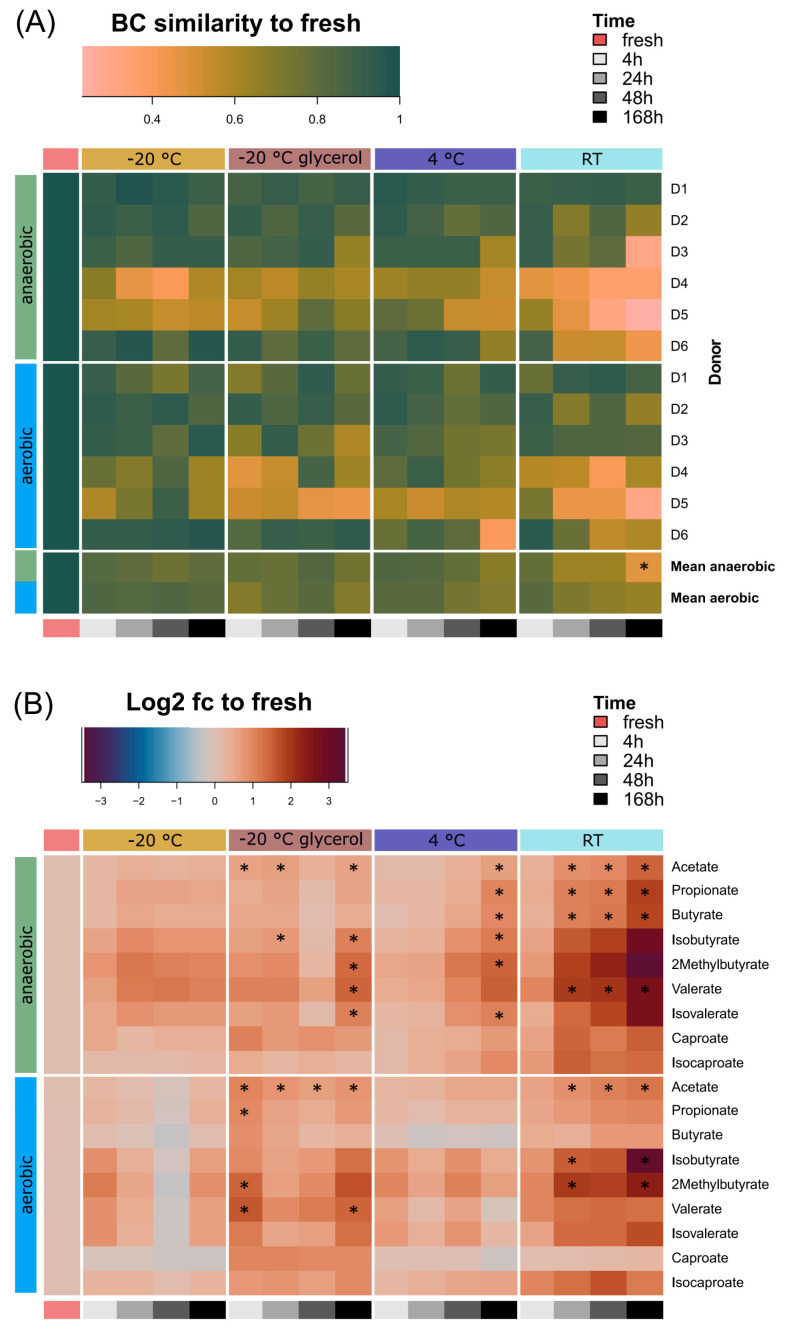
Changes in SCFA profiles and concentrations. (**A**) Heat map visualization of BC similarity per SCFA profile compared to fresh, resolved for storage conditions and oxygen exposure, plotted individually per donor and as mean. (**B**) Heat map of log2-fold changes calculated using SCFA concentrations [µM]. Pairwise comparisons were calculated using Wilcoxon rank sum test for non-parametric data compared to 4 h time points in A and compared to fresh sample in B (n = 6). Asterisks indicate significance.

We then performed a detailed analysis of the different conditions to assess their impact on individual samples and identify suitable logistics preserving microbiota composition and functionality. SCFAs were identified and quantified by targeted mass spectrometry (Figure 5).

The BC similarity of each sample kept under different conditions was compared to that of the fresh sample resolved per donor and as the mean across donors (Figure 5A). Reflecting the high individuality of the donors, SCFA profiles showed donor-specific changes (Figure 5A). While BC similarity decreased significantly across all conditions (Supplementary Table S1), compared to the 4 h time point, only samples stored at RT for 168 h under anaerobic conditions showed significant changes (Supplementary Table S2). Nevertheless, BC similarity gradually decreased at RT and 4 °C, independent of oxygen exposure, but remained stable at −20 °C (Supplementary Table S2).

To determine changes within the SCFA profiles, we compared the absolute SCFA abundances [µM] as log2-fold changes to “fresh” samples (Figure 5B, Supplementary Table S4). Many SCFA levels increased over time under anaerobic and non-frozen conditions. At RT, the concentrations of acetate, butyrate, propionate and valerate increased significantly after 24 h, while at 4 °C, an increase in acetate, propionate, butyrate, isobutyrate, 2-methylbutyrate and isovalerate was observed after 168 h. Furthermore, glycerol significantly altered several SCFAs under both aerobic and anaerobic conditions, in particular acetate, isobutyrate, 2-methylbutyrate, valerate and isovalerate. SCFA concentrations remained stable for up to 48 h at 4 °C and up to 168 h at −20 °C without glycerol. Despite the observed changes in absolute SCFA levels, relative profiles remained similar to the fresh profiles across conditions and time (Supplementary Figure S6).

In summary, storage at 4 °C or freezing at −20 °C without glycerol best preserved SCFA profiles.

### 3.3. Global Metabolite Profiles Are Highly Sensitive to Sample Logistics

**Figure 6:**
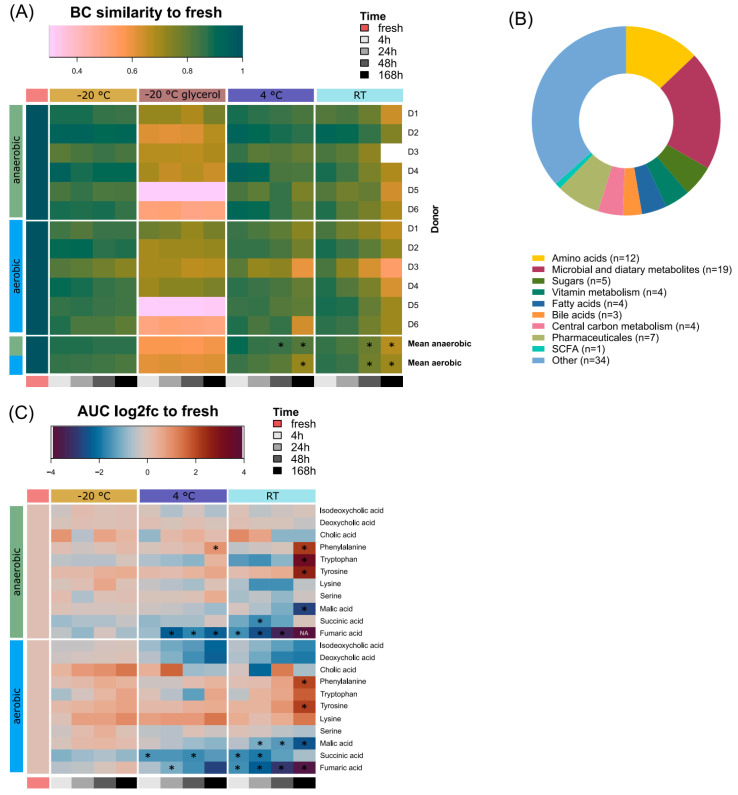
Changes in global metabolite profiles and metabolite abundances. (**A**) Heat map visualization of BC similarity per global metabolite profile compared to fresh, resolved for storage conditions and oxygen exposure, plotted individually per donor and as mean. (**B**) Classification of metabolites (total n = 93) identified in all fresh samples assigned to compound classes. (**C**) Heat map of log 2-fold changes calculated on metabolite abundances [AUC]. Pairwise comparisons were calculated using Wilcoxon rank sum test for non-parametric data compared to 4 h time points in (**A**) and compared to fresh sample in C (n = 6). Asterisks indicate significance.

The global metabolite profiles were significantly altered compared to the fresh samples already after 4 h, independently of any condition, indicated by the significantly lower BC similarity (Figure 6, Supplementary Table S1).

Compared to the 4 h samples, the global metabolome was significantly altered after 48 h or longer at 4 °C and RT both in the presence and absence of oxygen (Supplementary Table S2). Higher temperatures and longer storage times had a greater impact on the metabolome, reflected in significant alterations at 4 °C and RT. At −20 °C without glycerol, the metabolome remained stable. Glycerol dramatically affected the metabolome, especially for donors D5 and D6 (Figure 6A), leading to the exclusion of glycerol containing samples from downstream analysis.

We selected 93 metabolites that were detectable in the fresh samples across donors. These comprised different substance classes covering amino acids, bile acids, SCFAs and other microbial or dietary metabolites (Figure 6B). We tracked representative intestinal metabolites, such as bile acids, proteinogenic amino acids and the TCA cycle to assess the impact of sample logistics (Figure 6C).

We observed a temperature- and storage-dependent effect, particularly at 4 °C and RT. In particular, the measured aromatic amino acids tryptophan, tyrosine and phenylalanine increased over time, while malic acid, fumaric acid and succinic acid decreased, regardless of oxygen exposure. The bile acids isodeoxycholic acid and deoxycholic acid remained mainly stable under anaerobic conditions with a slight but not significant decrease in abundance under non-frozen aerobic storage. The same is true for cholic acid, which did not change significantly at all.

### 3.4. Sample Logistics Only Slightly Affect Microbiota Composition

**Figure 7:**
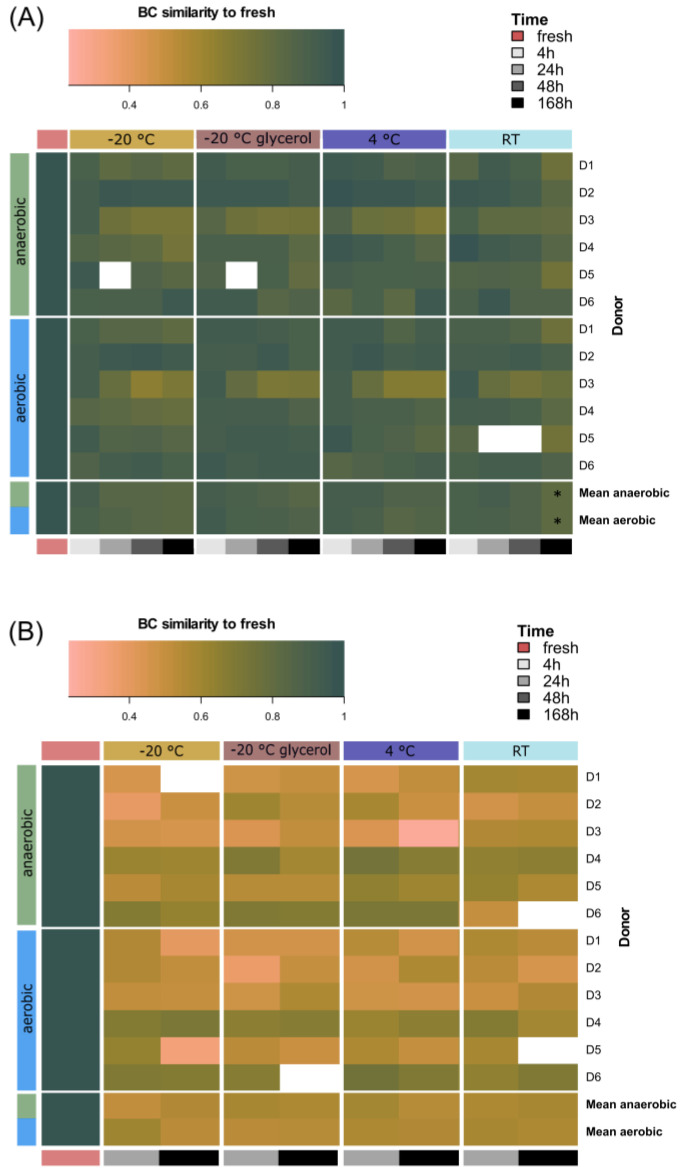
Changes in compositional profiles. (**A**) Heat map of BC similarities per MFC profile compared to fresh, resolved for storage conditions and oxygen exposure, plotted individually per donor and as mean. (**B**) Heat map of BC similarities per 16S rRNA profile (genus level) compared to fresh, resolved for storage conditions and oxygen exposure, plotted individually per donor and as mean. Pairwise comparisons were calculated using Wilcoxon rank sum test for non-parametric data compared to 4 h time points (n = 6). Asterisks indicate significance.

Lastly, we determined the effect of all storage conditions on faecal microbiota composition using MFC and 16S rRNA sequencing. We visualized the BC similarity of microbiota profiles to the fresh sample (Figure 7).

Donor individuality was already apparent in both cytometric microbiota profiles of the fresh sample (Supplementary Figure S7A) and species richness, i.e., number of observed species and Shannon diversity (Supplementary Figure S7B,C). The individuality was reflected in changes in BC similarity over time for both MFC (Figure 7A) and 16S rRNA sequencing (Figure 7B). The mean BC similarity of the first sampling time point across donors was significantly reduced compared to the fresh sample, i.e., 4 h for MFC and 24 h for 16S rRNA sequencing, respectively (Supplementary Table S1), suggesting a correlation of MFC and 16S rRNA-based taxonomic fingerprinting [28]. No significant changes in BC similarity or differential abundant genera were observed when comparing the fresh sample to the 4 h time point (Figure 7B, Supplementary Figure S8A), though individual microbiota profiles varied slightly (Supplementary Figure S8B). Species richness slightly increased after 168 h of aerobic storage at −20 °C with the related Shannon diversity failing to reach statistical significance (Supplementary Table S7).

With MFC, we only observed a significant change in BC similarity when comparing 168 h of aerobic and anaerobic storage at RT to the 4 h samples (Supplementary Table S2). Our data show that microbial composition generally remained stable under all conditions.

## 4. Discussion

To date, microbiome research has focused on sequencing-based methods, for which the impact of preservatives in the long- and short-term storage of faecal samples has already been investigated [9,10,24,29,40]. However, not only is the preservation of samples of interest for analysing microbiota composition, but the usability of samples for culturomics [41] or analyses of their metabolic capacity is also of increasing interest. Metabolites largely mediate host–microbiota interactions in health and disease [4]. Further, metabolites are subject to a comparably quick turn-over [42], rendering them especially susceptible to changes when transported or stored under sub-optimal conditions. Patients favour at-home sampling when enrolled in clinical studies [23] and may neglect using the home freezer/fridge for in between storage.

In this study, we aimed to allow for simple unsupervised home sampling and identify suitable logistics conditions that closely maintain the initial communities’ metabolite profiles alongside composition and taxonomy. We tested different storage temperatures, i.e., RT, 4 °C and −20 °C, over time (4 h, 24 h, 48 h and 168 h), both under aerobic and anaerobic conditions. These conditions were compared to samples immediately frozen and stored at −80 °C, neither affecting microbiota [43,44] nor metabolite composition [45]. Lately, the storage of faecal samples at −20 °C was demonstrated to be equally well suited to maintain the taxonomic composition while being energy- and cost-saving [40].

Cluster analysis highlights a very high donor-to-donor variability that dominated the effect of sample logistics, both in terms of microbiota functionality and composition. For microbial composition, this was expected, since a high donor individuality was already observed in the Human Microbiome Project (HMP) [46] and was proven in various later studies [10,47,48,49]. Although 16S rRNA sequencing and MFC were shown to correlate well [50,51,52,53], donor-specific clustering was more prominent in the cytometric profiles. For standard 16S rRNA sequencing, a defined sequence of the 16S rRNA gene is amplified, which allows for reliable taxonomic annotation to the genus level only. Thereby, the highly individual species- and strain-level information is lost. In contrast, MFC resolves the microbiota to the single-cell level [54], thereby preserving donor individuality. In terms of metabolites, we found the untargeted and SCFA metabolite profiles to also be highly donor-specific. Although in line with other metabolome studies [55,56], our findings are in contrast to the rather homogenous functionality predicted by [56]. Discrepancies may arise from the chosen level of functional analyses. Metagenomics and metabolomics analyses both identify microbial signatures in disease [55,57], thereby proving a considerable overlap between both analyses.

In our study, we quantified nine SCFAs and measured the global metabolome in an untargeted approach. Since SCFA biosynthesis represents an essential core function derived from dietary fibre fermentation [58], these compounds are omnipresent in the human gut. However, SCFAs only make up a minor part of the faecal metabolome. On the contrary, the global metabolome mirrors the enzymatic activity of the microbiota and further captures small molecules in an untargeted approach that originate from the host and diet [59], explaining the higher donor individuality. To circumvent donor individuality in our analyses, we determined the Bray–Curtis (BC) similarity of each donor’s microbiota to its fresh counterpart. Across all Omics and donors, the BC similarity was negatively correlated with time, indicating changes in composition and functionality. The strength of correlation was again individual, which was also reported by others [60,61]. Overall, the global metabolome was the most susceptible to increased temperatures and prolonged storage times, followed by the SCFA profiles and the cytometric microbiota profile, while the 16S rRNA profile remained largely unaffected. The high stability of native stool samples at RT and 4 °C for up to 72 h in sequencing-based analyses was described [9,24].

Contradicting findings exist regarding short-term sample stabilization. On the one hand, stabilization was shown to impact microbial taxonomy more than the transport of the native faecal sample [9], while on the other hand, the stabilization of faecal samples primarily in OMNIgene GUT was reported to be suitable for preserving microbial taxonomy [24]. In accordance with our results, the latter study showed a reduced effect on microbial taxonomy at 4 °C compared to RT. Across several studies, OMNIgene GUT stabilization proved to be the most suitable for sequencing-based analyses [8,29,62], while suitability for metabolomics remains contradicting [11,19]. We did not include a stabilizer in our study since sample stabilization may impact microbial viability as well as protein integrity and thus culture- or cell-based downstream analysis [17,63].

Oxygen exposure had only a marginal impact on all modalities. In line with our findings, it was reported that oxygen exposure does not alter microbial 16S rRNA profiles or high-nucleic acid cytometric profiles within 6 h. However, oxygen exposure significantly increased membrane permeability, indicating cell death in cytometric analysis [61]. Aerobic sample logistics and processing were shown to reduce obligate anaerobic microbes and beneficial butyrate producers numerically [60,64,65,66]. In the anaerobic environment, we observed more prominent effects on the SCFA profiles compared to those in the aerobic conditions. This may reflect the continued metabolic activity of anaerobic microbiota post-sampling when maintained at anaerobic conditions. Thus, depending on the planned usage of faecal microbiota samples, the maintenance of viable obligate anaerobes by continuing anaerobicity will have to be weighed against the accurate profiling of SCFA.

Before this study, limited data existed regarding the effects of temperature and time on faecal metabolites. In contrast to composition, microbial functionality was already affected during short-term sample logistics. The addition of glycerol influenced both the SCFA and global metabolite profiles, raising concerns about the advisability of using cryoprotectants when the research focus lies on the metabolome. In line with our results, faecal metabolites from feline microbiota were changed after only 6 h of storage at ambient temperature [67]. Gratton et al. observed metabolic shifts after 24 h at 4 °C, even earlier than our observed changes after 48 h [68]. While they focused on different metabolites using a ^1^H NMR spectroscopic analysis, they identified a similar trend of an overall change in the metabolome fingerprint and different stabilities for faecal metabolites in general.

Regarding SCFAs, Cunningham et al. reported a significant accumulation of SCFAs (acetate, butyrate and propionate) at RT within 24 h, which is consistent with our results. The maintenance of the samples at 4 °C reduced the accumulation of these SCFAs, but it did not prevent it [69]. Thus, for short-term storage and transportation, freezing at −20 °C is a suitable option. However, long-term storage (>4 weeks) at this temperature was generally questioned by De Spiegeleer et al. (2020), although different conditions may apply for the polar and nonpolar metabolome [20]. Our study was limited mainly to polar metabolites, which seems to be more sensitive to logistic-dependent alterations than nonpolar lipids [20].

## 5. Conclusions

The human faecal metabolome proved to be the most susceptible to sample logistics, especially when samples were stored chilled or at ambient temperatures (Table 1).

Storage at −20 °C resulted in the least change across all Omics, stabilizing both microbial composition and function, and thus may represent the most optimal condition for descriptive microbiota profiling. Sample logistics at ambient temperature for more than 24 h should be avoided. Thus, depending on the downstream analyses, sample logistics and storage should be adapted and carefully planned, especially in costly clinical or multi-donor studies.

## Figures and Tables

**Table 1:** Feasible sample logistic parameters for all Omics layer. ✓—no change observed, X—change observed after given time point.

Omics Layer	−20 °C	−20 °C Glycerol	4 °C	RT
16S	✓	✓	✓	✓
MFC	✓	✓	✓	X (>48 h)
SCFA	✓	X	X (>48 h)	X (>4 h)
Untargeted	✓	X	X (>24 h)	X (>24 h)

## Data Availability

The cytometric data that support the findings of this study are openly available in FLOW Repository under reference number FR-FCM-Z74S. 16S amplicon data are available on Sequence Read Archive (SRA) submission SUB14164451 with the project ID PRJNA1068571. SCFA data are available at the NIH Common Fund’s National Metabolomics Data Repository (NMDR) website, the Metabolomics Workbench, https://www.metabolomicsworkbench.org (accessed on 15 August 2024), where they were assigned Study ID ST003301 (SCFA). The data can be accessed directly via their Project DOI: https://dx.doi.org/10.21228/M8H521 (accessed on 15 August 2024) (SCFA).

## Supplementary Materials

The following supporting information can be downloaded at https://www.mdpi.com/article/10.3390/microorganisms12101998/s1. Figure S1: Rarefaction curves, Figure S2: Data processing for microbiota flow cytometry, Figure S3: Exemplary basepeak chromatograms, Figure S4: Bray-Crutis similarity for each time point, Figure S5: Donor-resolved correlation of Bray-Curtis similarity and time, Figure S6: Stacked bar plots of SCFA profiles, Figure S7: Individuality of the microbiota, Figure S8: 16S rRNA sequencing results. Results from statistical analyses are summarized on tables with Table S1: Wilxocon rank sum tes of Bray Curtis similarities per conditions—all Omics versus fresh, Table S2: Wilxocon rank sum test—Bray Curtis similarities per conditions—all Omics versus 4 h. Table S3: Wilxocon rank sum test—Bray Curtis similarities of taxonomic profiles (16S) versus 24 h, Table S4: Pairwise comparison of absolute SCFA abundances versus fresh per SCFA, Table S5: Pairwise comparison of relative SCFA abundances versus fresh per SCFA, Table S6: Pairwise comparison of identified metabolite abundances versus fresh metabolite, Table S7: Pairwise comparison of diversity indices versus fresh per index
