# Supplementary material for: Multi-Omics Analysis Unravels the Impact of Stool Sample Logistics on Metabolites and Microbial Composition
Source: Microorganisms. 2024 Sep 30;12(10):1998. doi: 10.3390/microorganisms12101998 (PMC11509235; doi:10.3390/microorganisms12101998)
Supplement: Supplementary file 1 [file microorganisms-12-01998-s001.zip › microorganisms-3186145-supplementary.docx]

**Multi-omics analysis unravel the impact of stool sample logistics on metabolites and microbial composition**

Jannike L. Krause^1†^*, Beatrice Engelmann^2†^, David J. D. Lallinger^1^, Ulrike Rolle-Kampczyk^2^, Martin von Bergen^2,3^, Hyun-Dong Chang^1,4^

^1^ German Rheumatism Research Center Berlin, a Leibniz Institute – DRFZ, Schwiete laboratory for microbiota and inflammation, Berlin, Germany

^2^ Helmholtz-Centre for Environmental Research - UFZ, Department of Molecular Toxicology, Leipzig, Germany

^3^ Institute of Biochemistry, Faculty of Biosciences, Pharmacy and Psychology, University of Leipzig, Germany

^4^ Chair of Cytometry, Institute of Biotechnology, Technical University Berlin, Germany

*** Correspondence:** Jannike L. Krause, [jannike.krause@drfz.de](mailto:jannike.krause@drfz.de)

**† equal authorship**

**Running title:** Multi-Omics analysis of clinical stool sample logistics

Supplementary Figures

**
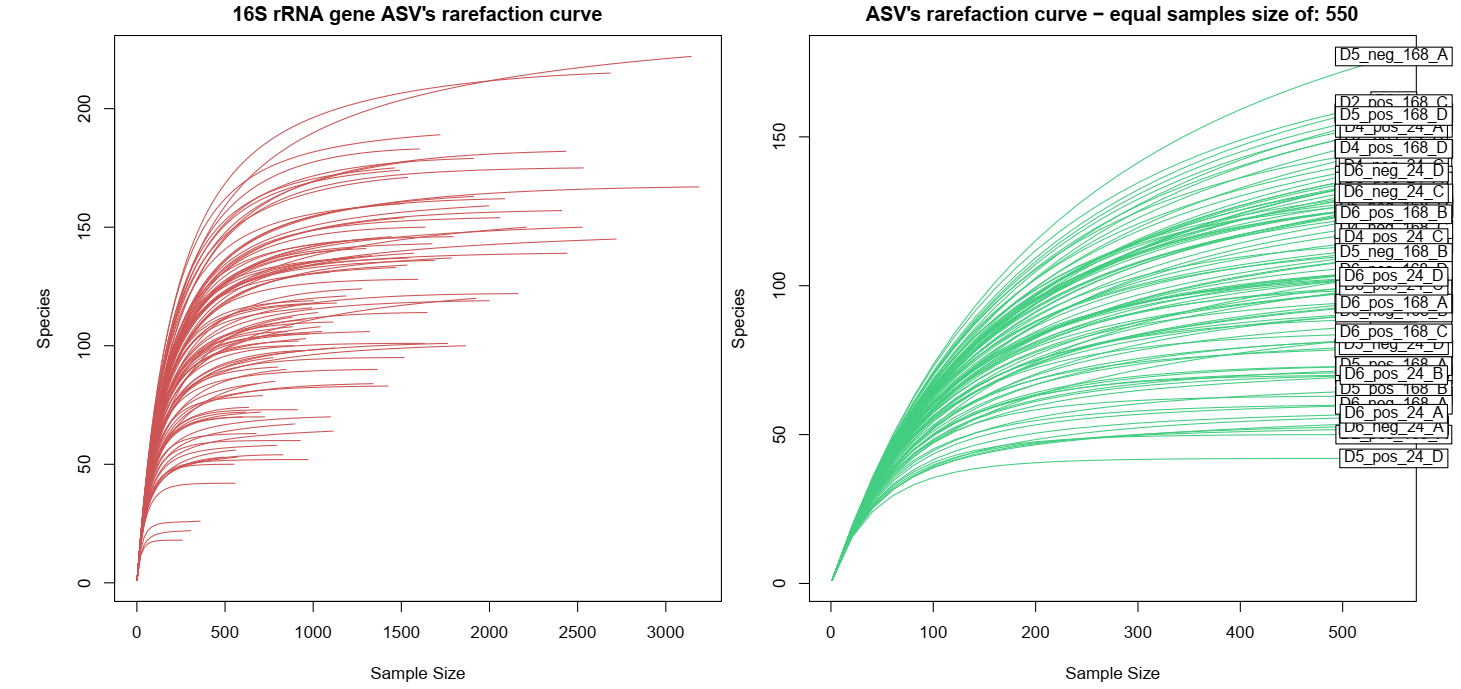
**

**Supplementary Figure S1.** Rarefaction curves. Rarefaction curves before (A) and after (B) rarefaction to equal sample size of 15,000 reads per sample


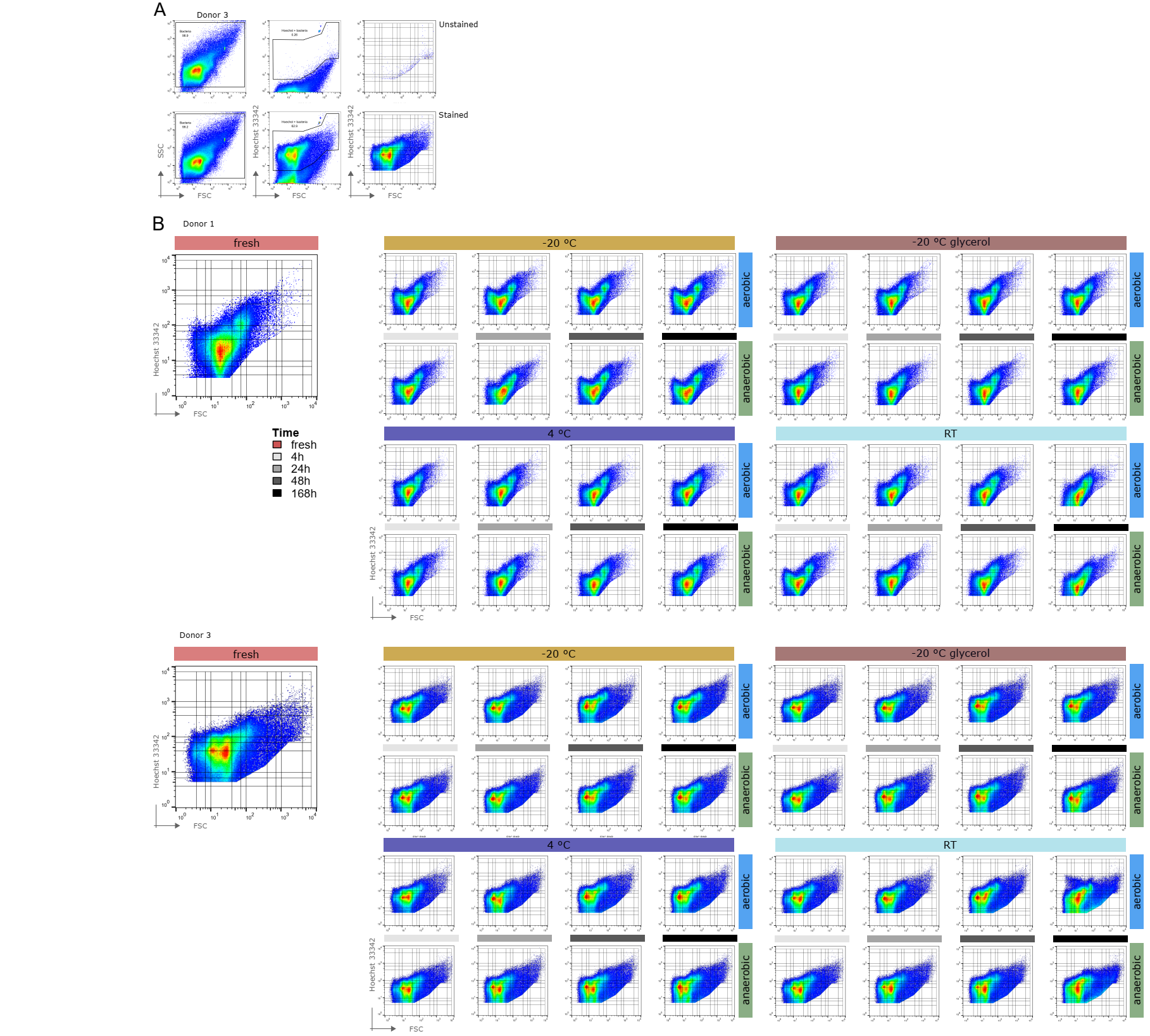


Supplementary Figure S2: Data processing for microbiota flow cytometry. (A) Gating strategy for faecal microbiota comprises selection of microbial events (left), identification of DNA high events (middle) and grid gating for comparison (left). (B) Exemplary dot plots of donor 1 and donor 3.


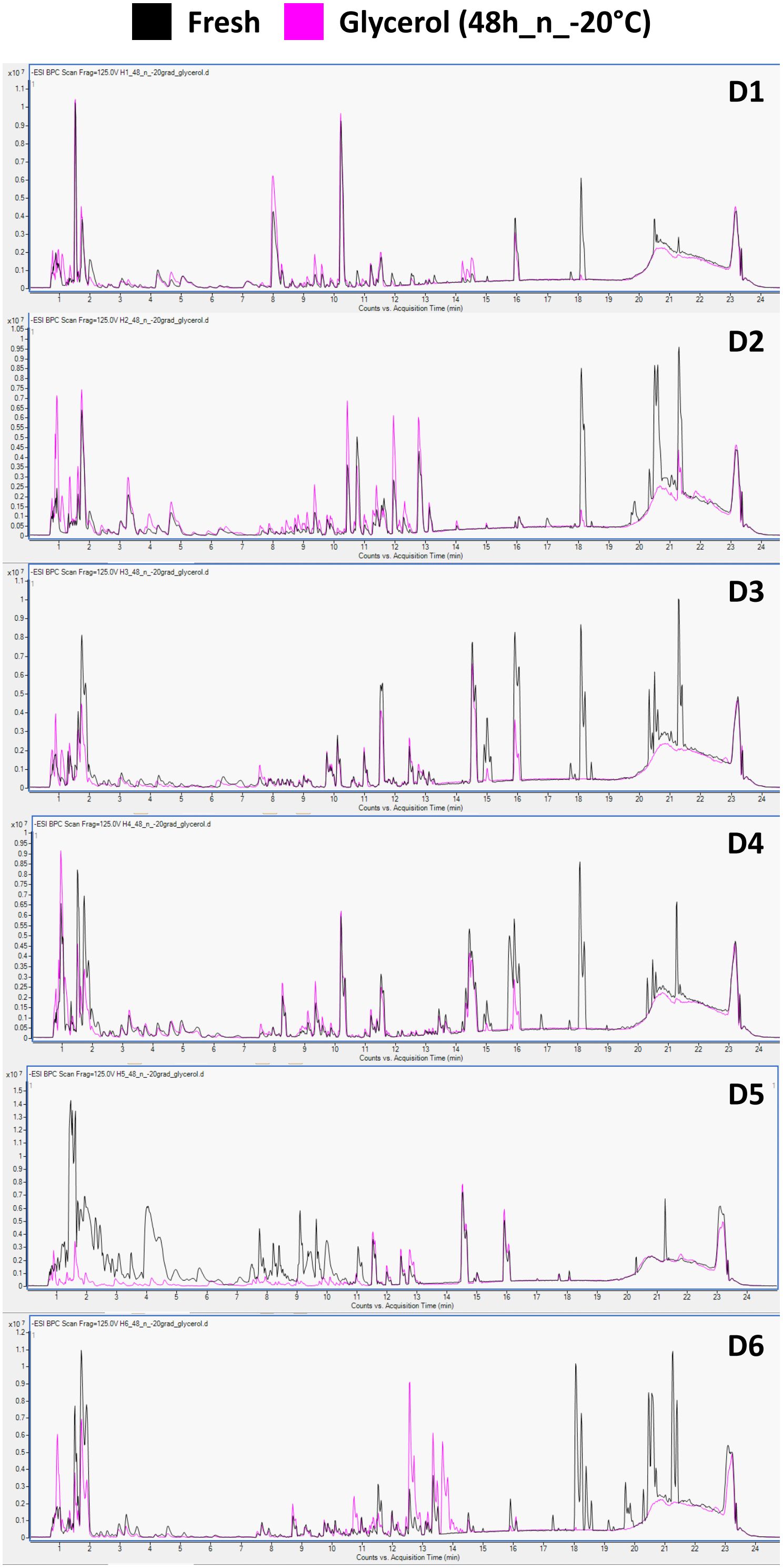


Supplementary Figure S3: Exemplary basepeak chromatograms. The basepeak chromatorgrams of the fresh sample (black) and a glycerol stored sample (pink, here: ‑20 °C, 48 h, anaerobic) were overlaid for peak comparison. One example per donor.


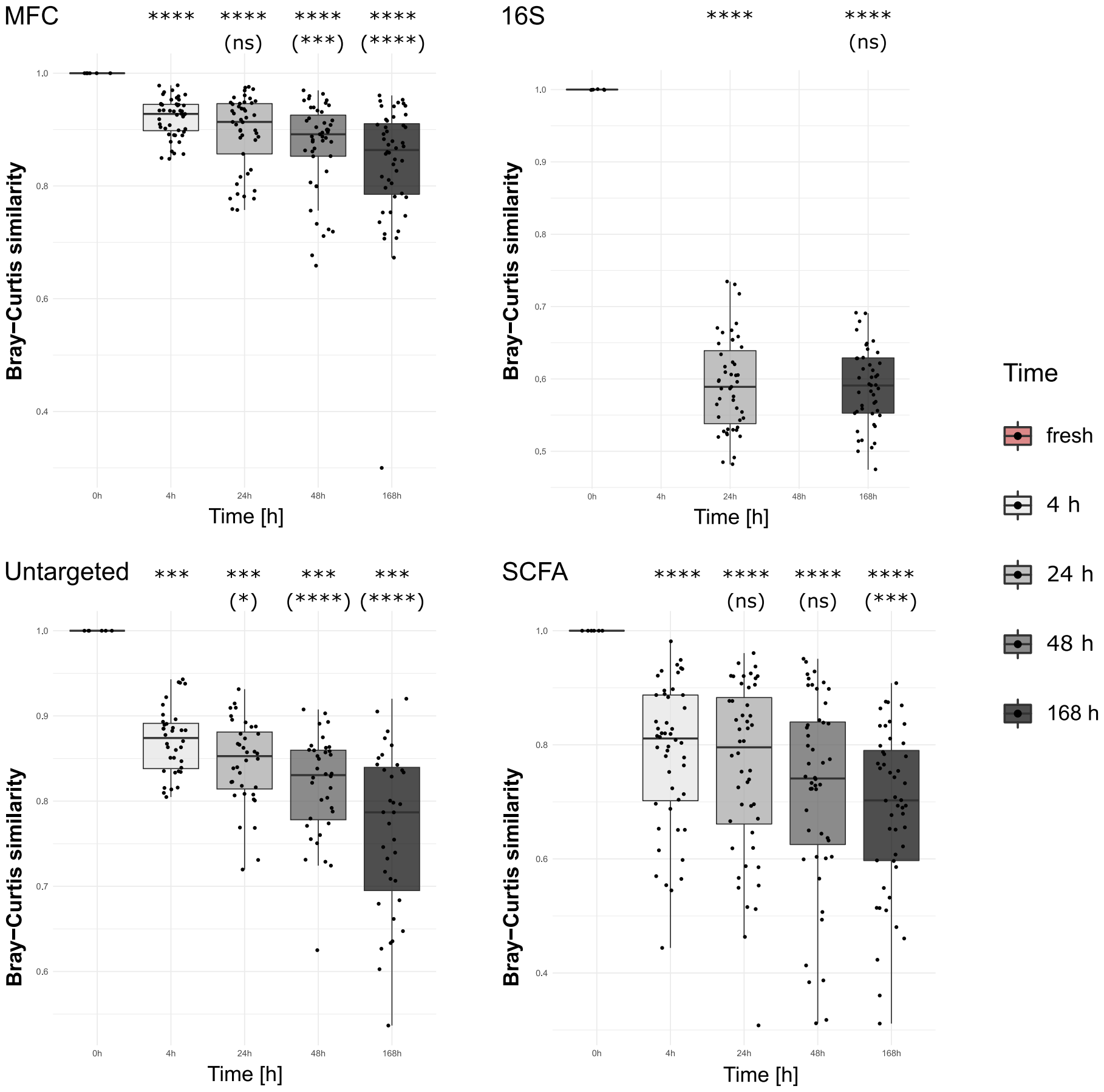


Supplementary Figure S4: Bray-Curtis similarity for each time point. The Bray-Curtis similarity of all samples was compared to the fresh sample per donor and plotted for each time point for the respective ‘Omics analyses, i.e. microbiota flow cytometry (MFC), 16S rRNA sequencing (16S), untargeted metabolomics (Untargeted) and SCFA profiling (SCFA). Asterisks indicate significance compared to the fresh sample, while asterisk in brackets indicate significance compared to the 4 h time point.


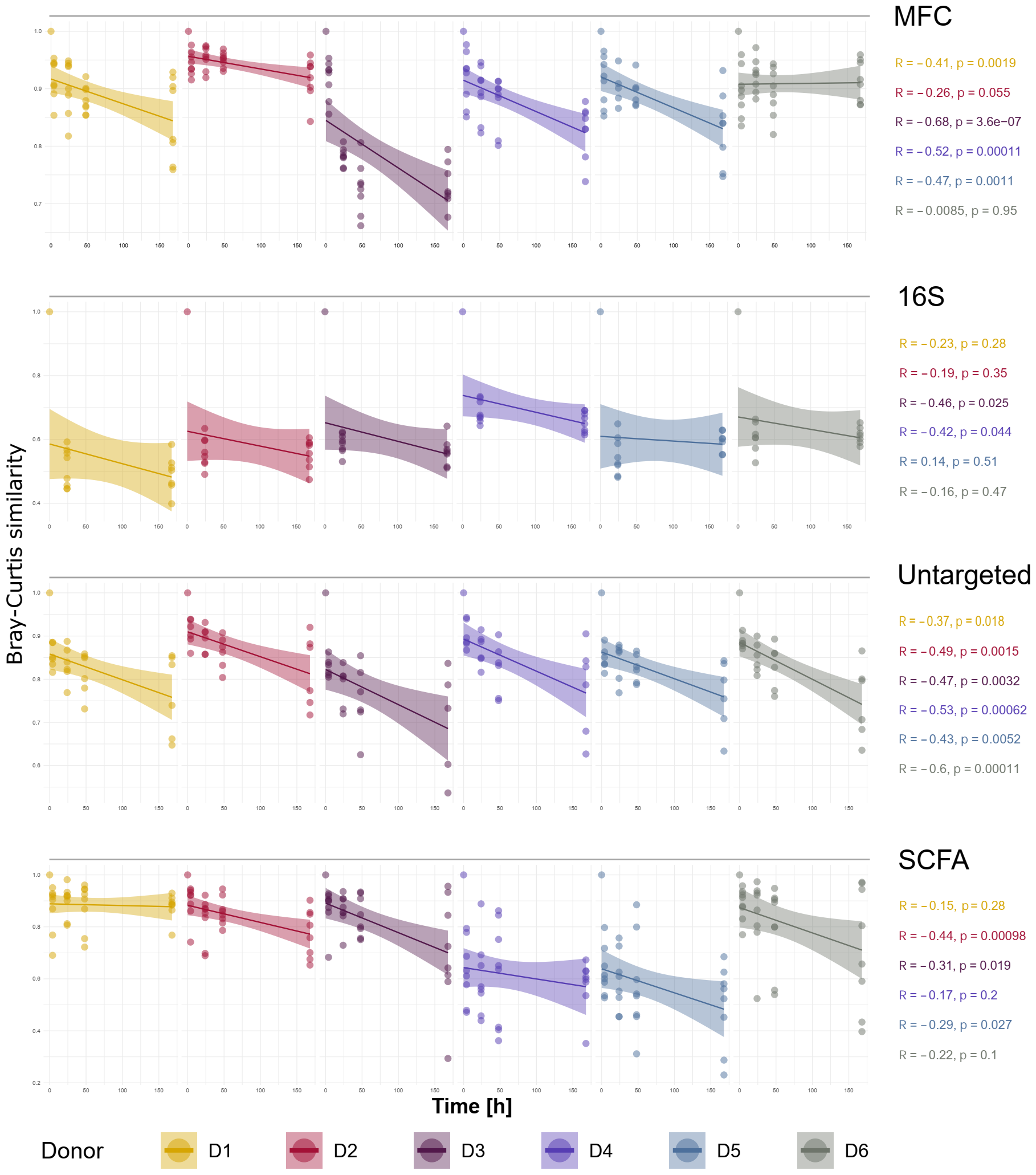


Supplementary Figure S5: Donor-resolved correlation of Bray-Curtis similarity and time. To quantify the association between the Bray-Curtis similarity and time, Kendall correlations were calculated for non-parametric data across all data points resolved per donor (D1-D6) and ‘Omics technique, i.e. microbiota flow cytometry (MFC), 16S rRNA sequencing (16S), untargeted metabolomics (Untargeted) and SCFA profiling (SCFA).

E.g., the microbiota composition of donor 3 assessed either by MFC or 16S was significantly and negatively correlated (R = -0.68, p = 3.6e-07, R = 0.46, p = 0.025, respectively), while there was barely any correlation between BC similarity and time for donor 6 in the same analyses (R = -0.0085, p = 0.95, R = -0.16, p = 0.47, respectively. Alike, the SCFA and untargeted metabolite profiles of donor 2 were most severely affected on the functional level (R = -0.44, p = 0.00098, R = -0.49, p = 0.0015, respectively), while the BC similarity of microbial functional profiles were less negatively correlated for other donors such as for instance donor 6.


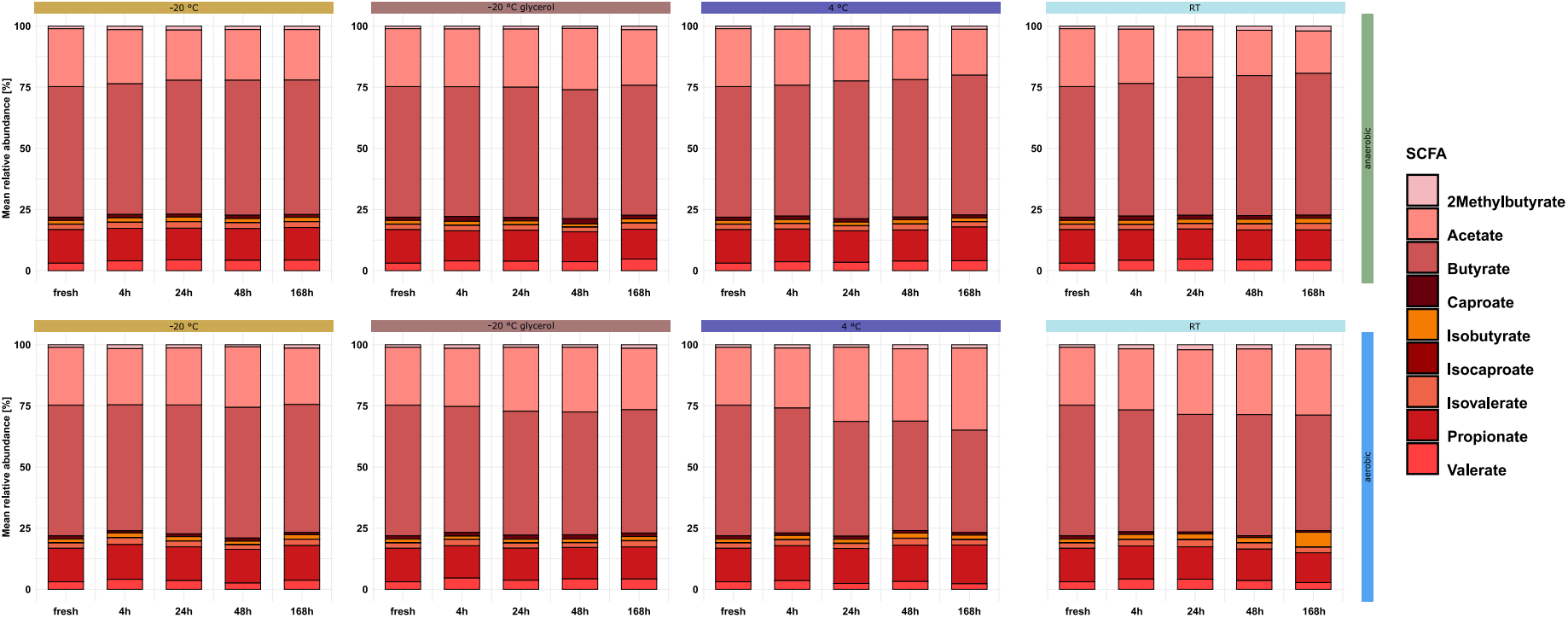


Supplementary Figure S6: Stacked bar plots of SCFA profiles. Mean relative SCFA abundances resolved by storage condition and oxygen exposure (n=6).


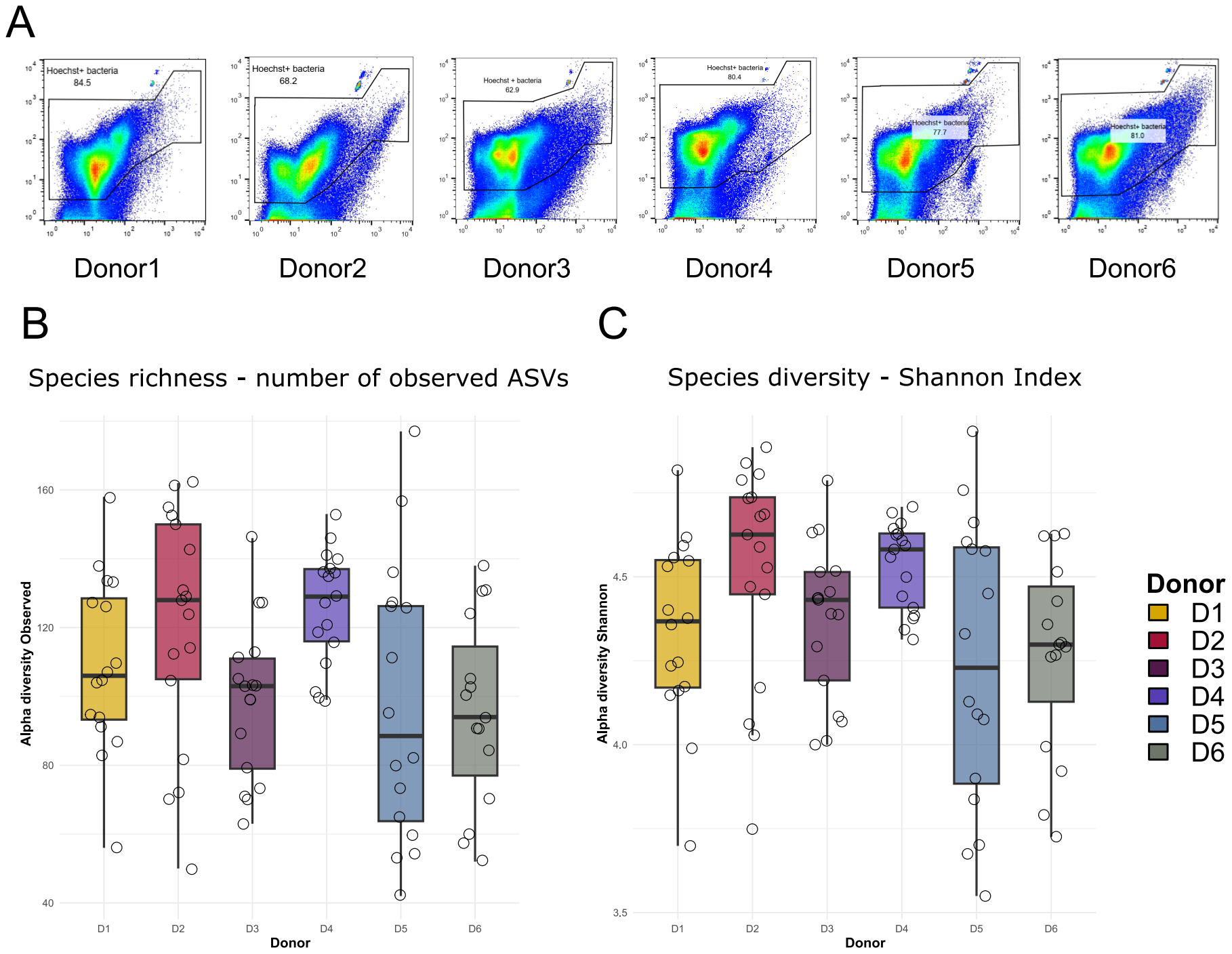


Supplementary Figure S7: Individuality of microbiota. (A) Cytometric profiles of fresh samples of each donor depicting microbiota structure by Hoechst staining and forward scatter properties. (B) Species richness as number of observed ASVs. (C) Species diversity quantified from Shannon diversity index.


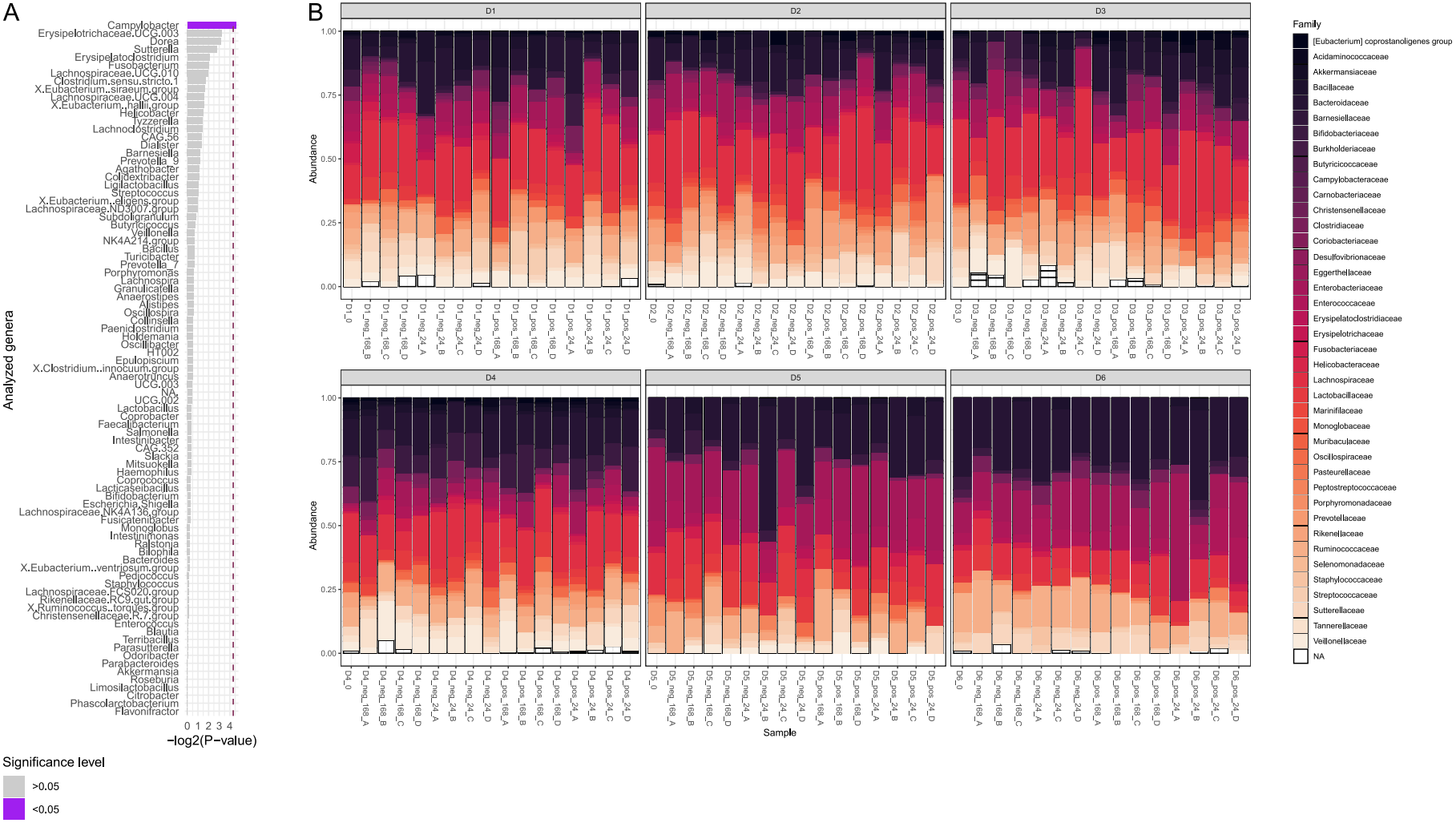


Supplementary Figure S8: 16S rRNA sequencing results. (A) Differential abundance analysis using Kruskal-Wallis rank sum test. (B) Family-level stacked bar plots of taxonomy per donor over all conditions.

.
